# Supplementary material for: Insecticidal Activity of Chitinases from Xenorhabdus nematophila HB310 and Its Relationship with the Toxin Complex
Source: Toxins (Basel). 2022 Sep 18;14(9):646. doi: 10.3390/toxins14090646 (PMC9505380; doi:10.3390/toxins14090646)
Supplement: Supplementary file 1 [file toxins-14-00646-s001.zip › toxins-1880943-supplementary.pdf]

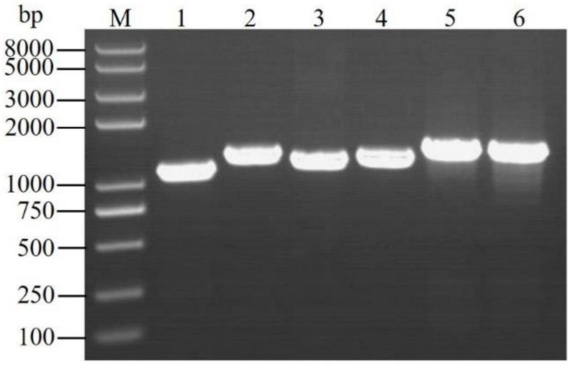

Figure S1. Amplification of *chi60*, *chi70*, *Kmr*, and *tetA* gene. Lane M, Trans2K plus Ⅱ DNA marker. Lane 1, the upstream fragment of *chi60* gene with a size of 1069 bp. Lane 2, the downstream fragment of *chi60* gene with a size of 1223 bp. Lane 3, the upstream fragment of *chi70* gene with a size of 1147 bp. Lane 4, the downstream fragment of *chi70* gene with a size of 1081 bp. Lane 5, the *Kmr* gene with a size of 1300 bp. Lane 6, the *tetA* gene with a size of 1300 bp.

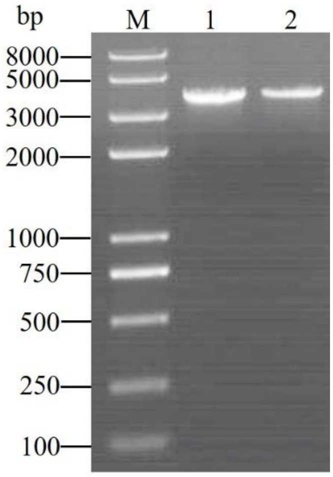

Figure S2. Amplification of fusion PCR. Lane M, Trans2K plus Ⅱ DNA marker. Lane 1, fusion products of the upstream fragment of *chi60* gene, downstream fragment of *chi60* gene, and *Kmr* gene. Lane 2, fusion products of the upstream fragment of *chi70* gene, downstream fragment of *chi70* gene, and *tetA* gene.

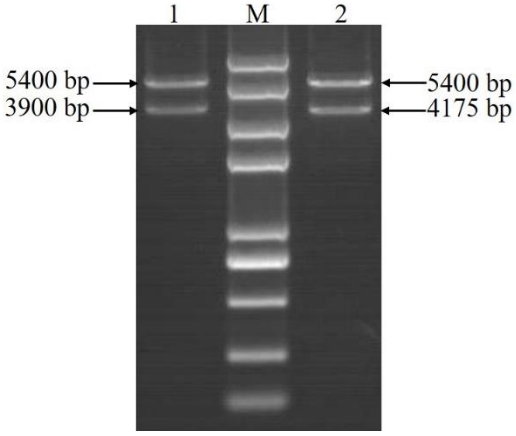

Figure S3. Double digestion of pJQ200SK-*chi60-Kmr* and pJQ200SK-*chi70-tetA* plasmids. Lane 1, pJQ200SK-*chi60-Kmr* plasmid digestion with *Xba* I/*Xho* I, the release of 5400 bp DNA fragment represented pJQ200SK vector while 3900 bp depicted the fusion fragment of *chi60-Kmr*. Lane M, Trans2K plus II DNA marker (8000, 5000, 3000, 2000, 1000, 750, 500, 250, and 100 bp). Lanes 2, pJQ200SK-*chi70-tetA* plasmid digestion with *Xba* I/*Xho* I, the release of

5400 bp DNA fragment represented pJQ200SK vector while 4175 bp depicted the fusion fragment of *chi70-tetA*.

Table S1. The sequence information of each motif.

Motif name	Sequences
Motif 1	BYEDSNADGKNGFTLYTDTVADADFLYNEESGHFISLDTPRTVKEKA EYVKEKGLGGIFIWSGDQDNGLLANAAREGLGY
Motif 2	JALSJGGWSMSGYFSVIAKDPEQRZTFVESJKDFFDRFPMFTDLDJDWE YPGSKGEEG
Motif 3	ELIAAGVDNJNVMTYDFFGTGWAEYJGHH
Motif 4	QNKYRYTSSKAMSDASDEIGEPLVAWQNQSGGQVWYVIFDSAVYKN TYWVERWHIPDRN
Motif 5	NEFDPDTSDSNLSYTPGRVAKNLFNEYESDPDF
Motif 6	TEPLPKVSAYITDWCQYDARLSPETQDNTALTSDDAPGRGFDLEKIPP TAYDRLIFSFMAVNGDKGKLSERINEVVDGWNRQAEASSGQIAPITL GHIVPVDPYGDLGTTRNVGLDADQRRDASPKNFLQYYNQDAASGLL GGLRNLKARA
Motif 7	FDRIIVGFAGIIGDEGLEEATINQAAIDFKIASDEDDLPNHKGEATFTD YWGDTGAYLNCGFPGWKETD
Motif 8	VEPKKIHJGYANYGRNAQGADJTTRSPNR
Motif 9	PRKEISIACSAVPAKLEKANI
Motif 10	VFGYJTDWGIYDSRYGNAPGD

Table S2. Amino acid sequences of chitinases used in phylogenetic analyses.

Strain name	Gene name	GenBank access	Sequences
X. nematophila HB310	chitinase 60	AGK44778	MPSSKNSNLNDNPFVIIPDPGTETKPGDIW GAGATEKTYEVNEFDPSTADSDLSYTPGRI AKNVFNHYESVSGFEVFGYLSDWGIYDSR YGNAPGDTDVDYGEGGRGTDIMRLLDEG SPLPYFDRIIVGFAGIIGDEGLKEATINQAAI DFKIASDEDDLPNHKGEATFTDYWGDTG AYLNCGFPGWKETDFTPENAQGVLGALV KLHKKYPDMPIGLSLGGWSMSQAFHFIAQ EPELRQRLAQSLKKIFDLFPMFTDLDLDWE YPNYKGEEHNSYDEEDPENFAELIKEIRKE LPDITISIATIAVPAGLEAANIPLLLEAGVD KLNVMTYDFFGTPWAETLGHHTALKLNP DKEETQNSVDKAVNYLLDELHVEPKKINI GYAGYTRNAQQASIPSISPIYGRYTPRGDIA LGSFESGSTEWPDLLRNYLDSNMDGINNF TVYTDEVAKAEFLYNQESRLFMSLDTPYSV KEKAQYVKEKGLGGMFIWMIDHDNGLLT NAAREGLGAATVGTPRVDMAPLCLSAAE KAKNRTK

Xenorhabdus nematophila HB310	chitinase 70	AGK44779	MSQNVYRYPSIKAMSDASSEVGASLVAW<br>QNQSGGQIWYVIYDSAVFKNIGWVERWH<br>IPDRNISSDLPVYENAWQYVREATPEEIAD<br>HGNPNTPDVPPGEKTEVLQYDALTEETYQ<br>KVGYKPDGSGTPLSYSSARVAKSLYNEYEV<br>DPENTEPLPKVSAYITDWCQYDARLSPET<br>QDNTALTSDDAPGRGFDLEKIPPTAYDRLI<br>FSFMAVNGDKGKLSERINEVVDGWNRQA<br>EASSGQIAPITLGHIVPVDPYGDLGTTRNV<br>GLDADQRRDASPKNFLQYYNQDAASGLL<br>GGLRNLKARAKQAGHKLELAFSIGGWSM<br>SGYFSVMAKDPEQRATFVSSIVDFFRRFPM<br>FTAVDIDWEYPGATGEEGNEFDPEHDGP<br>NYVLLVKELREALNIAFGTRARKEITIACS<br>AVVAKMEKSSFKEIAPYLDNIFVMTYDFFG<br>TGWAEYIGHHTNLYPPRYEYDGDNPPPP<br>NPDRDMDYSADEAIRFLLSQGVQPEKIHL<br>GFANYGRSCLGADLTTRRYNRTGEPLGTM<br>EKGAPEFFCLLNNQYDAEYEIARGKNQFE<br>LVTDTETDADALFNADGGHWISLDTPRTV<br>LHKGIYATKMKLGGIFSWSGDQDDGLLA<br>NAAHEGLGYLPVRGKEKIDMGPLYNKGR<br>LIQLPKVTRRKS
X. nematophila	chitinase	KHD28148	MPSSKNSNLNDNPFVIIPDPGTETKPGDIW<br>GAGATEKTYEVNEFDPSTADSDLSYTPGRI<br>AKNVFNHYESVSGFEVFGYLSDWGIYDSR<br>YGNAPGDTDVDYGEGGRGTDIMRLLDEG<br>SPLPYFDRIIVGFAGIIGDEGLKEATINQAAI<br>DFKIASDEDDLPNHKGEATFTDYWGDTG<br>AYLNCGFPGWKETDFTPENAQGVLGALV<br>KLHKKYPDMPIGLSLGGWSMSQAFHFIAQ<br>EPELRQRLAQSLKKIFDLFPMFTDLDLDWE<br>YPNYKGEEHNSYDEEDPENFAELIKEIRKE<br>LPDITISIATIAVPAGLEAANIPLLLEAGVD<br>KLNVMTYDFFGTPWAETLGHHTALKLNP<br>DKEETQNSVDKAVNYLLDELHVEPKKINI<br>GYAGYTRNAQQASIPSISPIYGRYTPRGDIA<br>LGSFESGSTEWPDLLRNYLDSNMDGINNF<br>TVYTDEVAKAEFLYNQESRLFMSLDTPYSV<br>KEKAQYVKEKGLGGMFIWMIDHDNGLLT<br>NAAREGLGAATVGTPRVDMAPLCLSAAE<br>KAKNRTK
Xenorhabdus nematophila	chitinase	KHD28152	MSQNVYRYPSIKAMSDASSEVGASLVAW<br>QNQSGGQTWYVIYDSAVFKNIGWVERW

			HIPDRNISPDLPVYENAWQYVREATPEEIA<br>DHGNPNTPDVPPGEKTEVLQYDALTEETY<br>QKVGYKPDGSGTPLSYSSARVAKSLYNEY<br>EVDPENTEPLPKVSAYITDWCQYDARLSP<br>ETQDNTALTSDDAPGRGFDLEKIPPTAYD<br>RLIFSFMAVNGDKGKLSERINEVVDGWNR<br>QAEASSGQIAPITLGHIVPVDPYGDLGTTR<br>NVGLDADQRRDASPKNFLQYYNQDAAS<br>GLLGGLRNLKARAKQAGHKLELAFSIGG<br>WSMSGYFSVMAKDPEQRATFVSSIVDFFR<br>RFPMFTAVDIDWEYPGATGEEGNEFDPEH<br>DGPNYVLLVKELREALNIAFGTRARKEITI<br>ACSAVVAKMEKSSFKEIAPYLDNIFVMTY<br>DFFGTGWAEYIGHHTNLYPPRYEYDGDN<br>PPPPNPDRDMDYSADEAIRFLLSQGVQPE<br>KIHLGFANYGRSCLGADLTTRRYNRTGEP<br>LGTMEKGAPEFFCLLNNQYDAEYEIARGK<br>NQFELVTDTETDADALFNADGGHWISLD<br>TPRTVLHKGIYATKMKLGGIFSWSGDQDD<br>GLLANAAHEGLGYLPVRGKEKIDMGPLY<br>NKGRLIQLPKVTRRKS
Xenorhabdus vietnamensis	chitinase	OTA14562	MSSSKNTNLNNNPFVIIPDPGTETKPGEIW<br>GAGATEKTYEVNEFDPSTADSDLSYTPGRI<br>AKNLFNHYESVSGFEVFGYLSDWGTYDSR<br>YGNAPGDTNVDYAKGGRGTDIMRLLDEG<br>SPLPYFDRIVVGFAGIIGDEGLEEATINQAA<br>IDFKIASDKDDLPNHKGEATFTDYWGDTG<br>AYLNCGFPGWKETDFTPENAQGVLGALV<br>KLREKYPDTPIGLSLGGWSMSQAFHFIAKE<br>SELRQRLAKSLKKIFDLFPMFTDLDLDWEY<br>PNYKGEEHNSYDEEDPDNFAELIKEIRKEL<br>PDITISIATIAVPAGLEAANIPLLLEAGVDK<br>LNVMTYDFFGTPWAETLGHHTALKLNPD<br>KEETQNSVDKAVNYLVDELHIEPQKINIG<br>YAGYTRNAQQASILSISPIYGRYTPRGNIAL<br>GSFESGSTEWPDLLRNYLDSNMDGINGFT<br>VHTDEVAKAEFLYNEESRLFMSLDTPYSV<br>KEKAKYVKEKGLGGMFIWMIDHDNGLLT<br>NAAREGLGAATVGTPRIDMAPLCLSAAE<br>KAKNKTK
Photorhabdus asymbiotica	chitinase	RKS65952	MVNKYTYTSSKSMSDVSDIIGEPLTAWKN<br>QAGGQVFNVIFDSGVYTNTYWVERWHVP<br>EPSSKDGTPHNAWKYLRPATADEIKQHG<br>NPTDGSVNPTEDIPSPVLQSDEITEKTYQRP

			EINFKPDGSGGNLAYTATRVCRPMYNEYE<br>TDKSKPKLSAYITDWCQYDARLDGHDEK<br>ADDRGRGFDLSTINVIAYDKLIFSFLGICGD<br>VGVKKDKINEVWQGWKDQGGNITEGHI<br>VPLDPYGDLGTARNVGLPEESANTDIGPG<br>TFLPYYQQKRASGLLGGLRELQKTARLAG<br>HKLELAFSIGGWSMSGYFSVMAADVALRS<br>VFVGSIVDFFERFPMFSCVDIDWEYPGSAG<br>EVGNVISDKDGENYALLIKELRESLDRRFG<br>REERKEISIACSGVKAKLATSNIAELVKNG<br>LDNIYLMSYDYFGTGWAPYIGHHTNLYSP<br>KDPDPLAETDLSAEVAINYLHQDLGIPLEK<br>IHLGYANYGRAGKGANLETREYNKAGDA<br>LGTMEKGSPEFFDIVNNYLDTEHTLATGK<br>SGFVLMTDTNADADFLFSEKEGHFISLDTP<br>RTVKQKAEYVAKNKLGGIFSWSGDQDCG<br>LLANAAREGMGYIAKSNDETIDMGPLYN<br>PGKPYYLKSIGEMKKSSD
Photorhabdus asymbiotica	GH18 family chitinase	RKS65955	MSKNESKTDSQLVYETDPHEDAGEGAAQ<br>KTYRLNGFDPKTADSTLSYTPTRLAKTVFN<br>TYEEKDDFNVLCYLSDWSIYDERLAPIND<br>DSFKIKGGRGADLMRFKGDKKEGKPFKRII<br>FSFAGIIGDPGEKKTTIIAASGENGWKMGK<br>DEQDILENHEGKPIPIDPWADVAAYLNCG<br>FTKWAGDPVDLYHQDKAQGVLGGLRLL<br>KEENPDLEISVSVGGWSMSGAFYKVCRDE<br>KLRQRFVEGIKDLYSKFDMLTHLDLDWEY<br>PGSAGEGNQFDKDDYKYFIELIKDLKNAN<br>ISNLKGISIAASADKAKMEAAHIPELIAAG<br>VNEINLMTYDFFTLGDGKLSHHTNLYRNK<br>DDEYSKYSVDDAVKYLIDLGVDRKLIYIGY<br>SGYTRNARTAELESKDNEQLVGKYTDGTS<br>TVGSFEHSVIEWTDIIYNYIDYENQIGRNGF<br>EVFHDQIAKADYIYNKELKVFMSLDTPRS<br>VREKGRYVKENGLGGLFIWSGDQDNGLL<br>TNAAHEGLGRKVIKKVIEMKPFYFEGELPS<br>YDKPKEKQCEACKLNLFF
Xenorhabdus szentirmaii	chitinase	PHM35498	MSSSKNSSLKDNQFVIIPDPGTETKPGDIW<br>GAGATEKTYEVNEFDPSTADSDLSYTPGRI<br>AKNVFNHYESVSGFEVFGYLSDWGIYDSR<br>YGNAPGDTDVDYSEGGRGTDIMRLLDEGS<br>SLPYFDRIIVGFAGIIGDEGLNEETINQAAI<br>DFKIASDESDIPNHIGAATFTDYWGDTGA<br>YLNCGFPGWKETDFTPENAQGVLGALVK

			LHKKYPNMPIGLSLGGWSMSQAFHFIAKE<br>PDQRQRLAQSLKKIFDLFPMFTDLDLDWE<br>YPNYKGEEHNCYDETDPEHFAELIKDIRE<br>VLPDITISIATIAVPDGLKAANIPLLLEAGV<br>DKLNVMTYDFFGTPWAETLGHHTALKLN<br>PDKEKTQNSADKAVSYLLDELHVDPKKIN<br>IGYAGYTRNAQQASIPSISPIYGRYTPRDNI<br>ALGSFESGTTEWPDLLRNYLDSNMDGING<br>FTVYTDEVAKAEFLYNQESRLFMSLDTPYS<br>VKEKAKYVKEKGLGGMFIWMIDHDNGLL<br>TNAAREGLGATTVGTPRVDMAPLCLSAA<br>EKAKNRAK
Xenorhabdus szentirmaii	chitinase	PHM44313	MSQHVYRYPSVKAMSDASGEVGTSLVAW<br>QNQSGGQTWYVIYDSAVFKNIGWVERW<br>HIPDRNISPDLPVYENAWRYVRDATPEEIA<br>AHDNPSTPSVPPGDKTVILQYDELTEKTYQ<br>EVGYKPDGSGTPLSYSSARVATSMYNEYE<br>GDPEITEPLPKISAYITDWCQYDARLSPETQ<br>GNAALDSDNAPGRGFDLEKIPPTAYDRLI<br>FSFMAVNGDKGKLSERINEVVVGWNRQA<br>EASSGQIAPITPGHIVPVDPYGDLGTTRNV<br>GLNADQRRDAGPQNFLQYYHQEAASGLL<br>GGLRNLKGRAKRAGHKLELAFSIGGWSM<br>SGYFSVMAKDPAQRTTFVNSVIDFFRRFPM<br>FTAVDIDWEYPGASGEEGNEFDPENDGPN<br>YVLLVKELREKLDIAFGTRARKEITIACSAV<br>VAKMEKSSFKEIAPYLDNIFVMTYDFFGTG<br>WAEYIGHHTNLYPPKYEYDGDNPPPPNP<br>DREMDYSADEAIRFLLSQGVQPEKIHLGF<br>ANYGRSCLGADLATRRYNRTGEPLGTME<br>KGAPEFFCLLNNQYDAEYALSRGKNQFEL<br>VTDTETDADALFNADGGHWISLDTPRTVL<br>HKGIYAAKMKLGGIFSWSGDQDDGLLAN<br>AAHEGVGYLPVPGKEKIDMGPLYNKGRLI<br>QLSKVTRSKP
Photorhabdus luminescens	chitinase	PQQ37426	MTDNRKYQYTCKKAMSDASENIGAPLTA<br>WSNQSGGETYYVIFDGQVYKNTYWVERW<br>HIPERNNIPSYENAWLWVREATAEEIANH<br>GNPKEGTVEPIPGDVAILKPDAITEQTYQE<br>KGYKPDGSGTNLSYTSARVCHSLYNPYET<br>DKTRPKVSAYITDWCQYDARLPSNKDKD<br>EDDGPGRGFNLADIPPTAYDRLVFSFLGIY<br>GDKGEKSEKINLSAEGWNKQLKPEDPPITF<br>GHIVPVDPYGDLGTTRNVGLPEEDKRDAG

			PNTFLQYYNQQAASGLLGGLRNLQQQAKLAGHRLELAFSIGGWSMSGYFSPMLKDTVQRKTFIDSIVDFFQRFPMFTAVDIDWEYPGTIGADGNEYDEINDGPNYAILIKELRQALDRAFGTSARKQITIASSAVVGKLKKSNIKELIRNGLDNIFVMTYDFFGSGWAEYIGHHTNLYSPDYASEDPDRLYDLSADEAIKYLIEVEGVPPGKIHLGFANYGRSCVGADLKTRLYNRNGQALGTMENGAPEFFCLLNNQFDCEQQLAWAKNGFKLMTDTATDADFLYNSTGGHFISLDTPRTVFKKGIYATEHKLGGIFSWSGDQDCGLLANAAREGAGYIPIKGKEKIDMGPLYNRGELVELPDVNKK
Photorhabdus luminescens	chitinase	PQQ40172	MADKKVPQDDGNLYISPDPGTKDLKEPEKVWGAGAAEKTYAANKFDPATSDKQLSYTPGRVVKNLFNNYQANPDFQVFGYLTDWGIYDSRYGEFAGDTNVDYTIGGRGTDIMRLKHDNYPRYDKIIVGFAGIIGDEGSEKDSINKGAVDFAIAQDAEDLINHRGKVTFIDAWADVQAYLNCGFAGWVPGDPPEMFDPNKAQGVLGALVKLTKVAKPPKVGLSLGGWTMSQAFHHIAKEPESRESLAESLKKIFDTFPMFTDLDLDWEYPGVEGAPGNDYGDEDAANYALLIEAVKQKVPHIKISIALNADPKKMEKANVLELIKAGVEGLNVMSYDFFGSPWAETLMHHTNLKRDPANNELNSMEDAVNYLLGLNVNPKMIFCGFAAYSRNAQQAFVTQVSPLKGFYTPYDTDPEEDNTFGSFAEGVTEFPDLLRHYLDKNLQGKNSFTLYTDKVSDADFLYNENNGAFLSIETPRTVHEKAKFVKEKGLGGLFIWTIDSDNGLLVNAAREGLGGTPISPVTVNMSKFYGVGKIKLSSQRRRTQFPFITKRKQRKMVIARKK
Yersinia entomophaga	Chi2	ABG33867	MVNKYTYTSSKAMSDISDVIGEPLAAWDSQVGGRVFNVIFDGKVYTNTYWVERWQVPGIGSSDGNPHNAWKFVRAATADEINKIGNPTTADVKPTENIPSPILVEDKYTEETYSRPDVNFKEDGSQGNLSYTATRVCAPMYNHYVGDKTKPKLSAYITDWCQYDARLDGGGSKEEERGRGFDLATLMQNPATYDRLIFSFLGICGDIGNKSKKVQEVWDGWNAQAPSLGLPQIGKGHIVPLDPYGDLGTARNVGLPPESADTSIESGTFLPYYQQNRAAGLLGGLRELQKKAHAMGHKLDLAFSIGGWSLSSYFSAL

			AENPDERRVFVASVVDFFVRFPMFSCVDI<br>DWEYPGGGGDEGNISSDKDGENYVLLIKE<br>LRSALDSRFGYSNRKEISIACSGVKAKLKKS<br>NIDQLVANGLDNIYLMSYDFFGTIWADYI<br>GHHTNLYSPKDPGEQELFDLSAEAAIDYL<br>HNELGIPMEKIHLGYANYGRSAVGGDLTT<br>RQYTKNGPALGTMENGAPEFFDIVKNYM<br>DAEHSLSMGKNGFVLMTDTNADADFLFS<br>EAKGHFISLDTPRTVKQKGEYAAKNKLGG<br>VFSWSGDQDCGLLANAAREGLGYVADSN<br>QETIDMGPLYNPGKEIYLKSISEIKSK
Yersinia entomophaga	Chi1	ABG33870	MEKEEKSNLIYDKDPGYVWDNKNECEGA<br>AEETYQELNYEPSISADKLTWTPTRLAKTV<br>FNTYEDDDDFNVLCYFTDWSQYDPRIINK<br>EIRDTGGRSADILRLNTPDGRPFKRLIYSFG<br>GLIGDKKYSADGNASIAVRLGVATDPDDA<br>IANHKGKTIPVDPDGAVLASINCGFTKWE<br>AGDANERYNQEKAKGLLGGFRLLHEADK<br>ELEFSLSIGGWSMSGLFSEIAKDEILRTNFV<br>EGIKDFFQRFPMFSHLDIDWEYPGSIGAGN<br>PNSPDDGANFAILIQQITDAKISNLKGISIA<br>SSADPAKIDAANIPALMDAGVTGINLMTY<br>DFFTLGDGKLSHHTNIYRDPSDVYSKYSID<br>DAVTHLIDEKKVDPKAIFIGYAGYTRNAK<br>NATITTSIPSEEALKGTYTDANQTLGSFEYS<br>VLEWTDIICHYMDFEKGEGRNGYKLVHD<br>KVAKADYLYSEATKVFISLDTPRSVRDKGR<br>YVKDKGLGGLFIWSGDQDNGILTNAAHE<br>GLKRRIKNKVIDMTPFYLDSDEELPTYTEP<br>AEPQCEACNIK
Photorhabdus temperata	chitinase	EQB98744	MKKKPHAADELYLSPDPGTKDLNEPDKV<br>WGAGAAEKTYAANEFDPATADKQLSYTP<br>GRVAKNLFNNYESDPDFLVFGYLTDWGIY<br>DSRYGEFAGDTDVDYTAGGRGSDIMRLK<br>DDNYPRYDKIIVGFSGIIGDEGEQQSNIEKG<br>AIDFAIAKDQDDLINHRGKATFLDGWAD<br>VQAYLNCGFAGWVPGDPPELFDPHKAQ<br>GVLGALVKLTQGAKPPKIGLSLGGWTMS<br>QAFHHIAKEPESRENLAESLKKIFDTFPMF<br>TDLDLDWEYPDVPGAEGNDYGDEDAENF<br>AELIKVIKQKLPDIKISIALNAEPGKMAKA<br>NVLELIKAGVEGLNVMCYDFFGSPWAEG<br>LAHHTNIKRDPNNDELNSMEDAVNYLLE<br>LNVNPKMIFCGFAAYSRNAQQAYVTKLSP

			LRGFYTPHDPDEDNTFGSFGPGVTEFPDLL<br>RNYLDSDLKGKNDFTLYTDTVSDADFLYN<br>ENTGAFLSIETPRTVHRKAEFVKNKGLGG<br>LFVWTIDADNGLLVNAAREGLGATPVAP<br>VTIDMSKFYHVGRAELSSQPRRSQPSFVTN<br>RHQSKTVFARKK
Photorhabdus khanii	chitinase	ETS30645	MVNKYTYTSSRSMSDVSDIIGEPLTAWKN<br>QAGGQVYNVIFNSGVYTNTYWVERWHIP<br>DPSSKEGTPHNAWKYIRPATEEEIKQHGN<br>PTDGSVNPTEDIPSPVLQPDDITEKTYQLP<br>DVNFKPDGSGNNLSYTATRVCRPMFNEY<br>ESDKSRPKLSAYITDWCQYDARLDGENEE<br>AGDRGRGFDLSTINITAYDKLIFSFLGIYGD<br>TGVKGKKIKEVAEGWNSQSDIKITEGHIVP<br>LDPYGDLGTARNVGLPQESANTDINSGTF<br>LPFYQQKKASGLLGGLRELQKRARLAGH<br>KLELAFSIGGWSMSGYFSVMAADVAQRR<br>VFVDSIVDFFERFPMFSCVDIDWEYPGSAG<br>EVGNVISEKDGEHYALLIKELRESLDSRFG<br>REARKEISIACSGVKAKLETSNIAELIKNGL<br>DNIFLMSYDYFGTGWAPYIGHHTNLYSPK<br>DPDASGASDLSAEVAINYLHQDLGIPLEKI<br>HLGYANYGRAGKGANLETRAYDINGNA<br>LGTMEKGSPEFFDIVNNYLDSEHTLATGK<br>NGFVLMTDTNADADFLFSEKEGHFISLDT<br>PRTVKQKAEYVAKNKLGGIFSWSGDQDC<br>GLLANAAREGMGYVAKSNDETIDMGPLY<br>NPGKFQYLKSIGEMKKNSD

Table S3. Primers used in this study.

Genes	Primer	Sequence (5′-3′)
chi60-up	60-up-F	CCG*CTCGAG*ACTGACTGTCAAGGTTTTTCC [1]
	60-up-R	CACGGCGCGCCTAGCAGCGGGCTAGATCTCCTTAATATGCG [2]
Kmr	Kmr-F	CCGCTGCTAGGCGCGCCGTGGGAGCCTATGGAAACTGGGAT [2]
	Kmr-R	GCAGGGATGCGGCCGCTGACACACCACAATATATCCTGCC [2]
chi60-down	60-down-F	GTCAGCGGCCGCATCCCTGCTCTCGGTTTATCCCAATCAG [2]
	60-down-R	GC*TCTAGA*CGCTGATTTCCCCTCTTTAT [1]
chi70-up	70-up-F	CCG*CTCGAG*AATCGGTGAAGGAATACGCATTGA [1]
	70-up-R	CACGGCGCGCCTAGCAGCGGAATTTCACTTCCGGCCCCGT [2]
tetA	tet-F	CCGCTGCTAGGCGCGCCGTGGTAATATTTACGTTGACACC [2]
	tet-R	GCAGGGATGCGGCCGCTGACATTACCCTGTTATCCCTACTAA [2]
chi70-down	70-down-F	GTCAGCGGCCGCATCCCTGCTAATAAAATTTCCGGTGGCCTC [2]
	70-down-R	GC*TCTAGA*GTTTCCTGACCATGAAGAGC [1]

[1] The underlined part represents the homologous arm sequence.
[2] The italicized part represents the enzyme cleavage sites.
